# Molecular arms races at the virus-host splicing interface and their pathogenic implications

**DOI:** 10.3389/fmicb.2026.1819878

**Published:** 2026-05-12

**Authors:** Yu Chen, Wenliang Pan, Xipeng Miao, Jinhua Liu, Zhimin Jiang

**Affiliations:** State Key Laboratory of Veterinary Public Health and Safety, Key Laboratory for Prevention and Control of Avian Influenza and Other Major Poultry Diseases, Ministry of Agriculture and Rural Affairs, College of Veterinary Medicine, China Agricultural University, Beijing, China

**Keywords:** alternative splicing, immune evasion, transcriptomics, viral circular RNA, virus-host interaction

## Abstract

RNA splicing is a fundamental driver of eukaryotic transcriptomic and proteomic diversity. Constrained by compact genomes, diverse DNA and RNA viruses, including adenovirus, HIV-1, and influenza virus, have evolved to hijack the host splicing machinery. This exploitation not only maximizes viral coding capacity but also ensures the precise spatiotemporal regulation of viral infection. In this review, we summarize current advances in the molecular mechanisms of viral RNA splicing, illustrating how viruses co-opt the host spliceosome and reprogram global alternative splicing landscapes to support their infection cycle. Through representative viral models, we detail the convergent strategies of alternative splice site selection and the dynamic interplay between viral RNA elements and host *trans*-acting factors. Furthermore, we spotlight the emerging frontier of viral circular RNAs (vcircRNAs), highlighting their biogenesis via non-canonical back-splicing and their versatile roles in immune evasion. Finally, we summarize recent methodological breakthroughs, particularly long-read sequencing and single-cell analyses, that are rapidly charting the complex splicing landscape. Together, this review provides an integrated perspective on the virus-host splicing interface, exposing critical vulnerabilities that offer promising avenues for next-generation, broad-spectrum antiviral interventions.

## 1 Introduction

The discovery of RNA splicing in Adenovirus serotype 2 in 1977 marked a watershed moment in molecular biology, overturning the “one gene, one polypeptide” dogma and revealing the discontinuous nature of eukaryotic genes (Berget et al., 1977; Chow et al., 1977a,b). At the time, while molecular biologists were accustomed to bacterial polycistronic operons yielding distinct proteins, eukaryotic splicing presented a radically different paradigm: enabling the production of multiple protein isoforms that share partial sequence identity yet exhibit profound functional differences (Marasco and Kornblihtt, 2023). This fundamental process, orchestrated by the spliceosome, involves the precise excision of introns and the ligation of exons (Wahl et al., 2009). The profound impact of alternative splicing (AS) is most evident in the scale of eukaryotic gene expression. Genome-wide analyses reveal that while the human genome comprises approximately 3.2 billion nucleotides, only ∼1.5% encodes roughly 20,000 protein-coding genes (Lander et al., 2001; Nurk et al., 2022). Remarkably, through AS, these limited genes give rise to an estimated 100,000 to 200,000 distinct proteins, representing a 5- to 10-fold expansion of proteomic diversity (Pan et al., 2008; Wright et al., 2022). Beyond its fundamental role in driving eukaryotic complexity, this extraordinary capacity for proteomic expansion makes RNA splicing an indispensable cornerstone of the viral life cycle. As obligate intracellular parasites constrained by compact genomes, viruses have evolved sophisticated strategies to hijack the host splicing machinery. By exploiting AS, viruses generate multiple functional isoforms from a single genetic template, thereby maximizing the output of their limited genetic information (Ashraf et al., 2019; Esparza et al., 2022). This dependency is remarkably conserved across diverse viral families, ranging from the segmented RNA genome of Influenza A Virus (IAV) to the DNA genomes of Hepatitis B Virus (HBV) and Human Papillomavirus (HPV) (Bogdanow et al., 2019; Kremsdorf et al., 2021; Wang et al., 2024). In these systems, alternative splicing functions not merely to diversify the viral proteome, but as a central regulatory switch that orchestrates the temporal progression of infection, governs the decision between viral latency and active replication, and modulates host-pathogen interactions.

Moreover, the landscape of viral RNA processing is far more complex than previously appreciated. Recent advances in long-read sequencing and specialized bioinformatics algorithms have uncovered that viruses utilize not only canonical linear splicing but also non-canonical “back-splicing” (Boldogkői et al., 2019; Fu et al., 2023; Toptan et al., 2018). This process generates viral circular RNAs (vcircRNAs), covalently closed transcripts that were historically dismissed as transcriptional noise but are now recognized as potent effectors in antagonizing host innate immunity and facilitating viral persistence (Dremel et al., 2025; Wang W. et al., 2023; Zhou et al., 2020).

Consequently, the virus-host splicing interface represents a critical frontier in virology, serving as both a determinant of pathogenesis and a vulnerability for therapeutic intervention. On one hand, targeting the splicing machinery with small molecules or antisense oligonucleotides offers a strategy to block viral replication (Li et al., 2022). On the other hand, the unique properties of back-splicing products, specifically the inherent biostability and immunogenicity of circRNAs, are being harnessed to develop next-generation antiviral vaccines (Liu et al., 2024; Qu et al., 2022).

In this review, we begin by providing a concise primer on the host splicing machinery before delving into how diverse viruses, from Adenovirus to HIV-1, exploit these mechanisms for proteome expansion. We then highlight how viral infection globally reprograms host alternative splicing to subvert innate immune surveillance. Furthermore, we explore the emerging paradigms of vcircRNA biogenesis and their roles in immune evasion. Finally, we examine the recent methodological breakthroughs charting the complex splicing landscape and discuss the dual potential of targeting viral splicing for therapy while repurposing splicing-derived circular RNAs as powerful biomedical tools.

## 2 Overview of RNA splicing: machinery and regulation

### 2.1 The spliceosome: composition and dynamic assembly

Pre-mRNA splicing is catalyzed by the spliceosome, a large and highly dynamic ribonucleoprotein (RNP) complex composed of five core small nuclear RNPs (snRNPs)-U1, U2, U4, U5, and U6-along with a complex network of over 300 non-snRNP auxiliary proteins (Fuxreiter et al., 2014; Wahl et al., 2009). Rather than existing as a preassembled entity, the spliceosome undergoes stepwise assembly on nascent transcripts (Yildirim et al., 2021).

**FIGURE 1:**
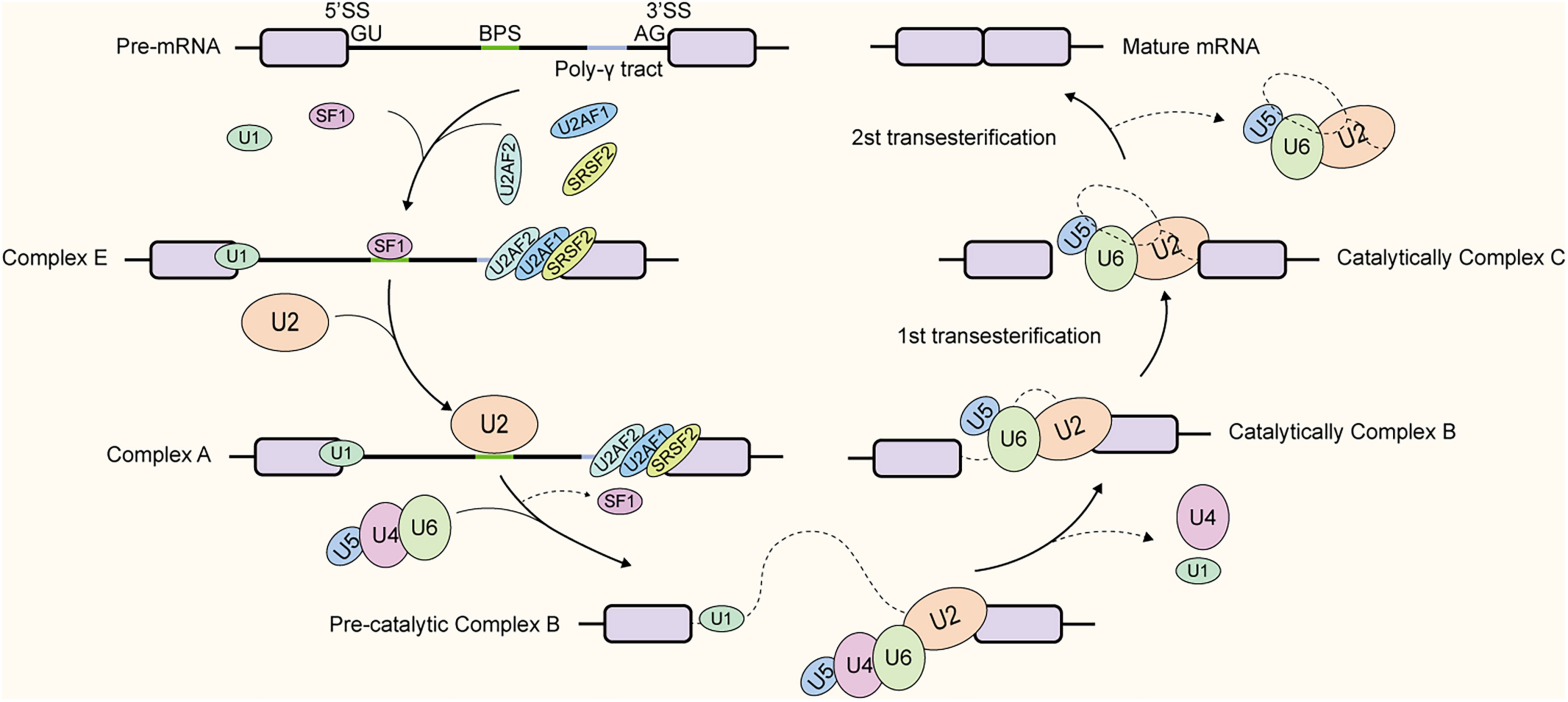
Stepwise assembly of the spliceosome. The assembly initiates with the formation of Complex E which U1 snRNP recognizes the 5′ splice site (5′SS) via base-pairing, SF1 binds to the branch point site (BPS), and the U2AF1 and U2AF2, which form a heterodimer, recognizes the 3′ splice sites (3′SS) and the polypyrimidine tract, respectively. Then, the U2 snRNP displaces SF1 at the BPS to form Complex A. Subsequently, the recruitment of the pre-assembled U4/U6. U5 to consist of the pre-catalytic Complex B. Next, extensive conformational rearrangements then trigger the release of U1 and U4 snRNPs, allowing U6 to connect to the 5′SS and U2 snRNP, and the activated spliceosome (Complex B) is generated. The first transesterification reaction generates Complex C, followed by further rearrangements that facilitate the second transesterification. Finally, the exons are ligated to form mature mRNA, the intron lariat is released for degradation, and the snRNPs are recycled for subsequent round of splicing.

This process is orchestrated by the recognition of four highly conserved sequence elements: the 5′ splice site (5′SS, GU), the branch-point (BP), the polypyrimidine tract (PPT), and the 3′ splice site (3′SS, AG) (Figure 1; Black, 2003). The BP, typically located 18–25 nucleotides upstream of the 3′SS, harbors the consensus motif (C/U)U(A/G)AC and contains a conserved adenine residue that is critical for the first step of splicing (Gao et al., 2008). Mechanistically, spliceosome assembly initiates with the base-pairing of the U1 snRNP to the 5′SS, followed by the recruitment of the U2 snRNP to the BP. Subsequent dynamic conformational rearrangements, involving the ordered assembly and dissociation of specific snRNPs, catalyze two consecutive transesterification reactions. These reactions result in the precise excision of the intron and the ligation of flanking exons to generate the mature mRNA (Zhan et al., 2022; Zhang Z. et al., 2024).

However, splice site selection is rarely a rigid process. Due to the presence of “Weak” or suboptimal splice sites in higher eukaryotes, spliceosome recognition exhibits remarkable plasticity (Wright et al., 2022). This inherent flexibility allows for the selective inclusion or exclusion of specific sequences from the same pre-mRNA precursor, a phenomenon known as alternative splicing (AS).

**FIGURE 2:**
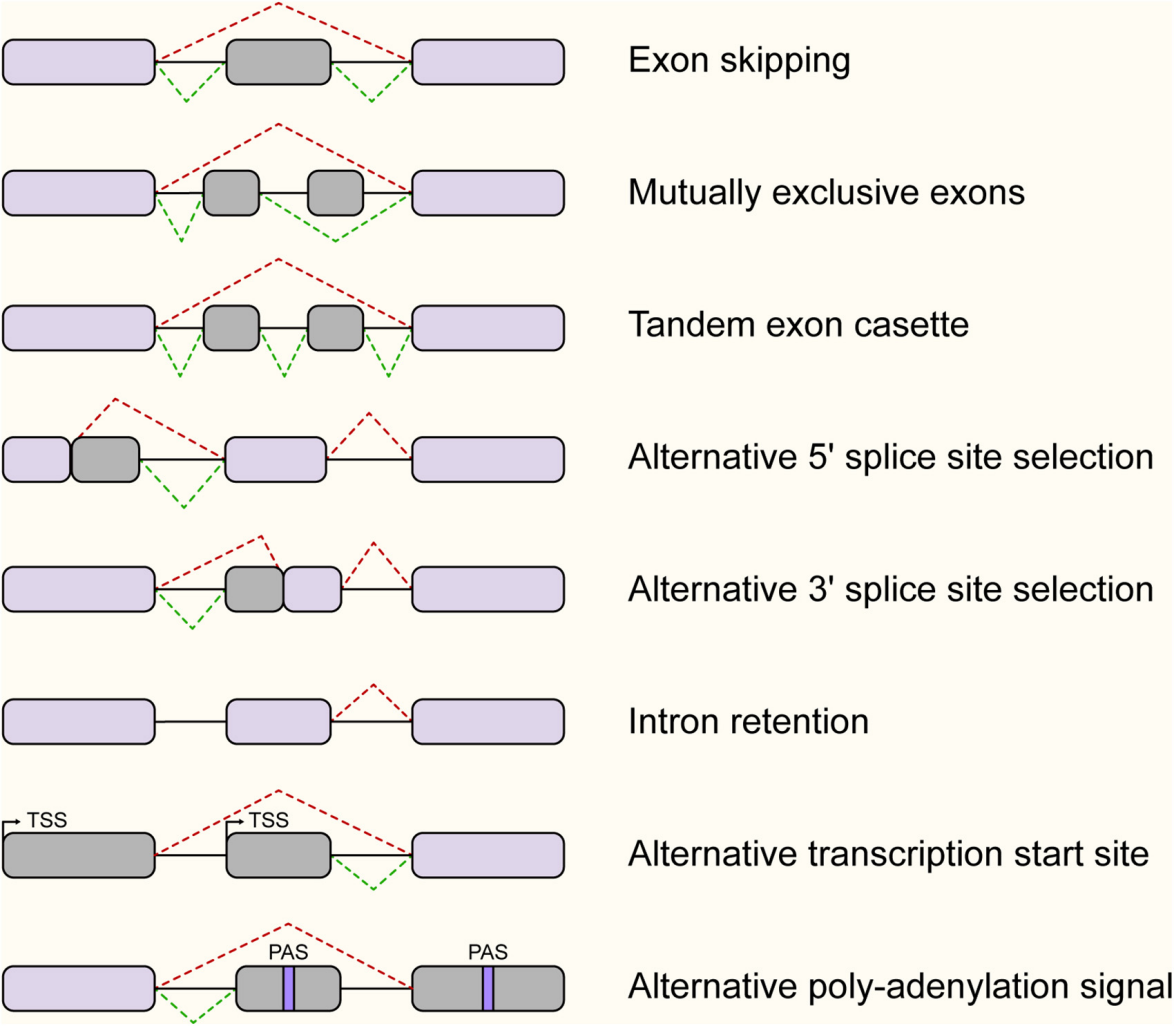
Major types of alternative splicing events. Schematic representation of distinct alternative splicing patterns. Exon Skipping: a cassette exon is either included or excluded from the mRNA. Mutually Exclusive Exons: one of two exons is retained, but never both. Tandem Exon Cassette: multiple consecutive exons are skipped or included together. Alternative 5′/3′ Splice Site Selection: usage of different splice sites extends or shortens an exon. Intron Retention: an intron is retained in the mature transcript. Also shown are Alternative Transcription Start Sites (TSS) and Alternative Polyadenylation Signals (PAS), which contribute to transcript diversity at the termini. Gray boxes represent constitutive exons; dark gray boxes represent alternative regions; blue lines represent introns.

This structural plasticity generates extensive transcript diversity through several canonical splicing patterns (Figure 2). The most prevalent mode in eukaryotes is Exon Skipping (SE), where a specific exon is either retained or excluded from the final mRNA. Other common modes include Mutually Exclusive Exons (MXE), where only one of two consecutive exons is selected, and Alternative 5′ (A5SS) or 3′ Splice Site Selection (A3SS), which alters the length of an exon by utilizing competing splice sites. Furthermore, Intron Retention (IR) involves the preservation of an intron in the mature transcript. The IR was not only predominant gene splicing mode in plants, but recent high-depth transcriptomic analyses have also revealed that it is highly prevalent and serves critical regulatory functions across mammalian systems and viral genomes (Boutz et al., 2015; Braunschweig et al., 2014). Beyond these splicing-specific events, transcript diversity is further expanded by Alternative Transcription Start Sites (TSS) and Alternative Polyadenylation (APA), which define the transcript’s 5′ and 3′ termini, respectively (Alfonso-Gonzalez and Hilgers, 2025). Consequently, the splicing outcome is not strictly hardwired but is dictated by a complex regulatory network.

### 2.2 Regulation of alternative splicing: *cis*-elements and *trans*-factors

**FIGURE 3:**
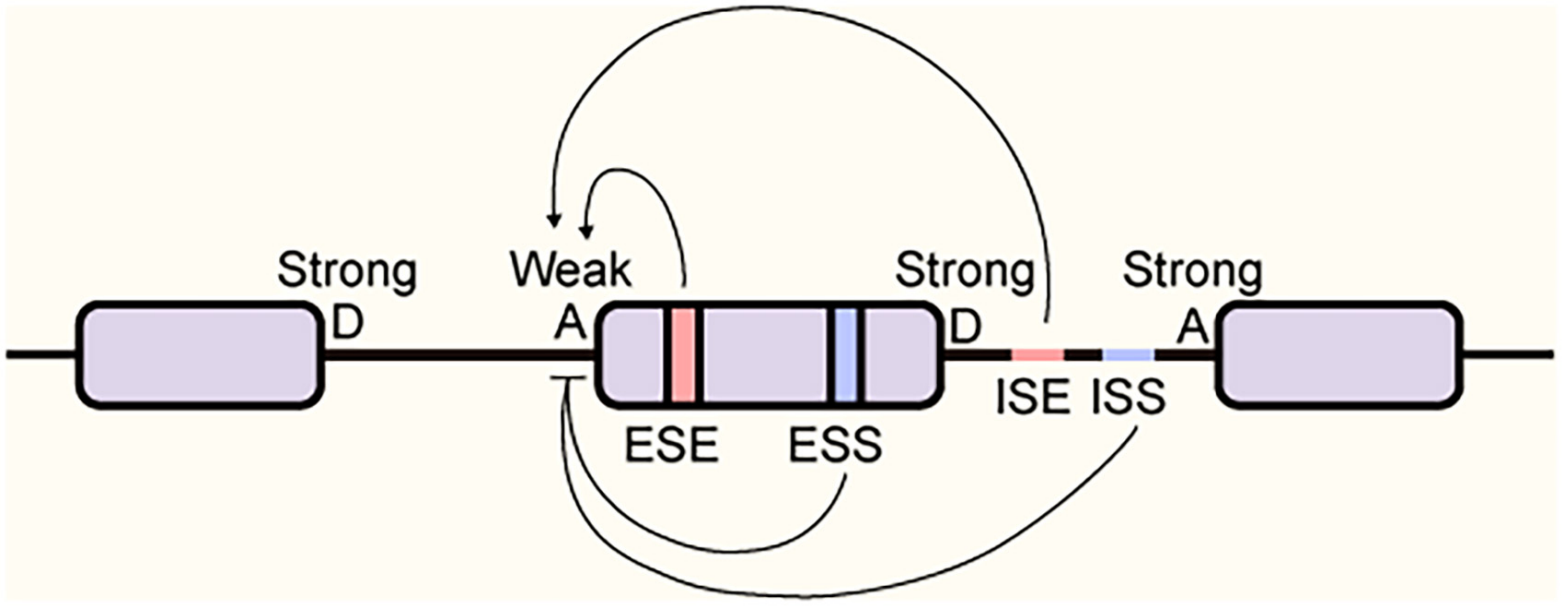
Regulation of alternative splicing by *cis*-regulatory elements. The schematic depicts a pre-mRNA segment containing an alternative cassette exon flanked by constitutive exons. The alternative exon is often defined by a “weak” or suboptimal 3′ splice site (Acceptor, A), rendering it dependent on auxiliary regulation. *Cis*-acting elements are classified based on their location and function: Exonic Splicing Enhancers (ESE) and Intronic Splicing Enhancers (ISE) recruit *trans*-acting factors to promote the recognition of the weak acceptor site and facilitate exon inclusion (indicated by arrows). Conversely, Exonic Splicing Silencers (ESS) and Intronic Splicing Silencers (ISS) recruit factors that repress splice site recognition and promote exon skipping (indicated by T-bars). D, Donor site (5′ splice site); A, Acceptor site (3′ splice site).

While the 5′ and 3′ splice sites define intron boundaries, these core sequences in higher eukaryotes are often degenerate and insufficient to distinguish authentic exons from the vast number of cryptic splice sites in the genome (Marasco and Kornblihtt, 2023). To compensate for this lack of specificity and enable fine-tuned regulation, pre-mRNAs have evolved a complex array of auxiliary *cis*-regulatory elements. Acting as “navigation markers” to guide spliceosome assembly, these elements are classified into four major categories based on their location and activity: exonic splicing enhancers (ESEs), exonic splicing silencers (ESSs), intronic splicing enhancers (ISEs), and intronic splicing silencers (ISSs) (Chen et al., 2021; Sette and Paronetto, 2022; Ule and Blencowe, 2019; Van Nostrand et al., 2020; Figure 3).

These cis-elements serve as binding platforms for trans-acting RNA-binding proteins (RBPs), and splicing outcomes are primarily dictated by the competitive interplay between two major antagonistic RBP families: the serine/arginine-rich (SR) proteins and heterogeneous nuclear ribonucleoproteins (hnRNPs) (Kim et al., 2021; Ouyang et al., 2022). Generally, SR proteins, exemplified by SRSF1 and SRSF2, function as splicing activators. By binding to enhancer elements, they recruit core spliceosomal components via their RS domains to facilitate exon inclusion (Li et al., 2022; Pengelly et al., 2022). In contrast, hnRNPs, most notably hnRNP A1 and PTBP1, typically act as repressors. They antagonize spliceosome assembly by binding to silencer motifs, where they induce steric hindrance, displace activators, or remodel RNA secondary structures (Chia et al., 2024; Miao et al., 2022; Zeng et al., 2023; Zhang Q. et al., 2021). Unlike constitutive exons that possess high-affinity splice sites, alternative exons rely on multiple low-affinity cis-elements. This degeneracy transforms splicing from a binary “on/off” switch into a highly tunable “rheostat.” Consequently, splicing outcomes are extraordinarily sensitive to subtle intracellular fluctuations in RBP concentration or phosphorylation status, which are often triggered by cellular stress or viral infection.

However, the canonical antagonism between SR proteins and hnRNPs represents a simplified model. In a physiological context, splicing regulation is a multidimensional process strictly modulated by the local genomic architecture, including RNA secondary structures that dictate splice site accessibility (Gao et al., 2022; Terai and Asai, 2022). Furthermore, splicing occurs co-transcriptionally, rendering it tightly coupled to RNA Polymerase II elongation rates and dynamic chromatin modifications (Uriostegui-Arcos et al., 2023). This regulatory complexity is further expanded by tissue-specific RBPs, which orchestrate splicing programs essential for establishing cell identity and differentiation (Ruta et al., 2024; Wang et al., 2022). Ultimately, while this profound molecular plasticity equips the host with the transcriptomic diversity necessary to adapt to environmental challenges, it simultaneously exposes a critical vulnerability: a highly tunable machinery that viruses readily exploit to maximize their own replicative fitness.

## 3 Exploitation of host machinery for viral proteome expansion

Viruses, particularly those constrained by compact genomes, confront a fundamental evolutionary bottleneck: severely limited coding capacity. To circumvent this physical constraint, they have evolved sophisticated strategies to maximize their genetic information density. By subverting the host’s AS machinery, often in synergy with co-transcriptional or translational recoding events, viruses can generate a remarkably diverse array of functional protein isoforms from a minimalistic set of primary transcripts. This paradigm of “molecular economy” effectively expands the viral proteome without the evolutionary cost of increasing genomic size. In the following subsections, we explore how this expansion strategy is masterfully executed across diverse viral classes, exemplified by DNA viruses like Adenovirus, and complex nuclear-replicating RNA viruses such as HIV-1 and Influenza A Virus (Li et al., 2022; Roesmann et al., 2024).

### 3.1 DNA viruses: mimicry of host genetic architectures for RNA splicing

#### 3.1.1 Adenovirus

Adenoviruses (AdVs), members of the Adenoviridae family, are non-enveloped double-stranded DNA (dsDNA) viruses with a linear genome of approximately 36 kb. These ubiquitous pathogens cause a broad spectrum of human illnesses, ranging from mild respiratory and ocular infections to severe gastroenteritis. Historically, Adenoviruses occupy a pivotal position in molecular biology, as the phenomenon of RNA splicing was first discovered during the analysis of Adenovirus 2 late mRNAs in 1977. This landmark discovery fundamentally reshaped eukaryotic gene expression paradigms and highlighted the virus’s absolute dependence on the host RNA processing machinery (Chow et al., 1977a,b; MacNeil et al., 2023).

**FIGURE 4:**
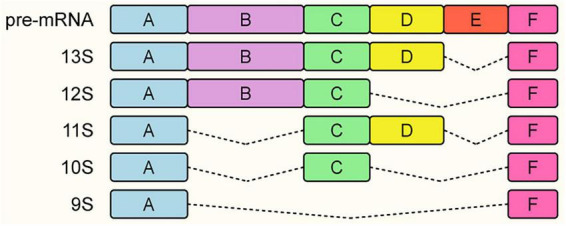
Schematic representation of Adenovirus E1A alternative splicing patterns. The E1A pre-mRNA undergoes differential splicing to generate multiple mRNA isoforms, including the major variants 13S, 12S, and 9S. The colored boxes represent exonic regions preserved in the mature transcripts, while the dashed lines indicate the intronic regions removed during splicing.

The viral *E1A* gene stands as the paradigmatic example of this splicing-dependent regulation. As the first transcriptional unit activated upon infection, the *E1A* pre-mRNA is differentially processed via competing alternative 5′ and 3′ splice sites to generate multiple isoforms, predominantly the 13S, 12S, and 9S mRNAs (Figure 4). Because these isoforms utilize alternative internal splice sites, the resulting proteins share identical N- and C-termini but diverge significantly in their internal regulatory domains. These structural variations dictate their distinct capacities to reprogram the host cell cycle-specifically by sequestering retinoblastoma (Rb) protein to drive S-phase entry and establish a cellular environment conducive to viral DNA replication.

Furthermore, AdVs brilliantly exploit splicing to orchestrate the temporal transition from early to late infection. During the late phase, a highly specialized, non-canonical splicing event appends a common 200-nucleotide non-coding sequence, known as the “tripartite leader,” to the 5′ end of all major late viral mRNAs (Svensson et al., 1983). This unique 5′ untranslated region (UTR) confers a massive translational advantage. By enabling viral transcripts to undergo ribosome shunting, the tripartite leader allows AdV mRNAs to efficiently bypass global host translational shutoff mechanisms, such as eIF2α phosphorylation, thereby ensuring the preferential synthesis of viral structural proteins during the final stages of the replicative cycle (Ulfendahl et al., 1985).

#### 3.1.2 Human Papillomavirus

Human Papillomaviruses (HPVs) are non-enveloped, circular double-stranded DNA viruses that rely heavily on host RNA processing to orchestrate gene expression from their highly compact ∼8 kb genomes. In high-risk genotypes such as HPV-16 and HPV-18, the primary etiological agents of cervical and oropharyngeal malignancies, alternative splicing acts as the master regulator of the viral oncoproteins E6 and E7 (Jin et al., 2024; Markowitz and Unger, 2023). The *E6* and *E7* genes are initially transcribed as a single bicistronic pre-mRNA. Crucially, within the full-length unspliced transcript, the downstream *E7* open reading frame (ORF) is translationally repressed. This suppression occurs because the minimal intercistronic distance severely hinders efficient ribosome termination and re-initiation (Wang et al., 2024).

**FIGURE 5:**
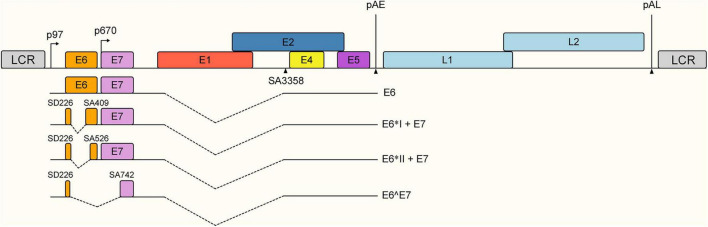
Schematic representation of HPV-16 E6/E7 alternative splicing transcripts. Transcription initiated from the early promoter p97 generates a polycistronic pre-mRNA spanning the E6 and E7 open reading frames (ORFs), where alternative splicing events determine the specific mRNA isoforms produced. The unspliced transcript remains intact in the E6 coding region, primarily encoding the full-length E6 oncoprotein. The E6*I + E7 isoform is generated by a splicing event connecting the splice donor site at nucleotide 226 (SD226) to the splice acceptor site at nucleotide 409 (SA409); this is the most abundant spliced isoform, producing a truncated E6 protein (E6I) and remodeling the transcript to allow efficient translation of the E7 oncoprotein. Other isoforms include E6II + E7, resulting from splicing between SD226 and SA526, and the E6∧E7 fusion transcript generated by splicing from SD226 to SA742. Key genomic elements, including the Long Control Region (LCR), promoters (arrows), and polyadenylation sites (pAE, pAL), are indicated.

To circumvent this translational bottleneck, the virus exploits specific splice sites (e.g., the SD226 donor and SA409 acceptor in HPV-16) to excise an intron embedded within the *E6* coding region. This precise splicing event serves a dual purpose: it generates truncated E6 isoforms (such as E6*I) while simultaneously remodeling the transcript architecture to unmask the *E7* initiation codon, thereby licensing it for efficient translation (Figure 5).

Consequently, the modulation of splicing efficiency directly dictates the relative abundance of viral early proteins. Unspliced mRNAs preferentially direct the synthesis of full-length E6 which mediates p53 degradation, whereas the spliced transcripts are an absolute prerequisite for the production of E7 which inactivates the retinoblastoma protein (pRb). Therefore, the delicate balance between intron retention and splicing excision strictly governs the E6/E7 ratio, a critical determinant of viral persistence, cell cycle deregulation, and ultimate oncogenic transformation (Jiang et al., 2024).

#### 3.1.3 Hepatitis B Virus

Hepatitis B Virus (HBV), the prototype of the Hepadnaviridae family, possesses one of the most compact genomes among human DNA viruses, spanning a mere ∼3.2 kb. Its replication cycle relies on an RNA intermediate, the pregenomic RNA (pgRNA), which canonically must remain unspliced to serve as the template for reverse transcription and to encode the essential core and polymerase proteins. Despite this stringent requirement for genomic integrity, HBV paradoxically co-opts the host splicing machinery to generate multiple spliced variants from the pgRNA precursor (Hsu et al., 2023; Nguyen et al., 2020).

**FIGURE 6:**
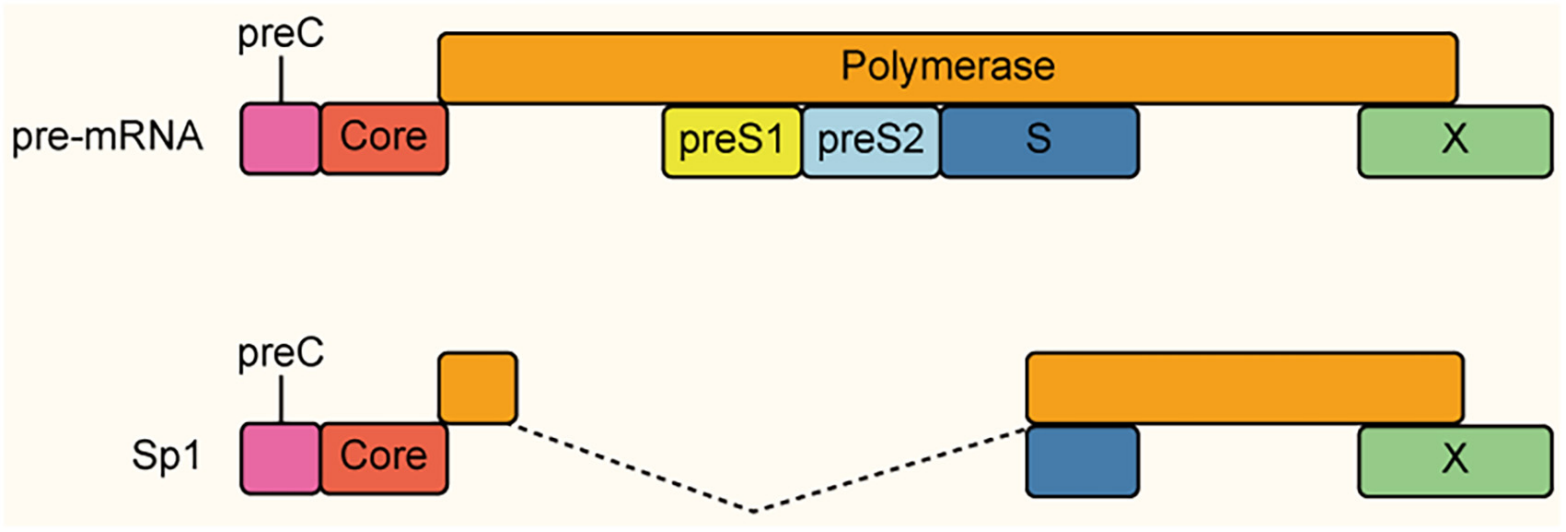
Schematic representation of the HBV Sp1 alternative splicing event. The pre-mRNA (top) represents the unspliced pregenomic RNA (pgRNA), which encompasses the open reading frames for viral Core, Polymerase, Surface (preS1/preS2/S), and X proteins. The Sp1 isoform (bottom) is the most abundant spliced variant, generated by the excision of a specific intron (indicated by dashed lines) that removes the C-terminal portion of the Core gene and the N-terminal region of the Polymerase gene (spanning the preS1 and preS2 domains). This splicing event fuses the splice donor site in the Core gene with a splice acceptor site, creating a fusion transcript that encodes a distinct chimeric protein known as the Hepatitis B Spliced Protein (HBSP), which is implicated in viral immune evasion.

The most abundant spliced isoform, Sp1, results from the excision of a specific intron spanning the C-terminal core and N-terminal polymerase genes (Figure 6). Translation of this transcript yields a distinct chimeric protein, the Hepatitis B Spliced Protein (HBSP). Unlike structural viral components, HBSP functions as a potent immunomodulatory factor (Luk et al., 2021). Specifically, HBSP interferes with host signaling to downregulate the chemokine CCL2, thereby attenuating the recruitment of monocytes and macrophages to the liver, a mechanism that severely blunts the innate immune response and facilitates long-term viral persistence (Duriez et al., 2017; Kremsdorf et al., 2021).

Clinically, the ratio of unspliced to spliced transcripts serves as a critical prognostic indicator. While full-length pgRNA drives active replication, the accumulation of spliced variants and HBSP strongly correlates with liver disease progression, fibrosis, and hepatocellular carcinoma (HCC). Thus, alternative splicing in HBV represents a fascinating evolutionary trade-off, intentionally diverting a fraction of its limited coding capacity away from direct viral replication to orchestrate pathogenesis and immune evasion (Candotti and Allain, 2017).

### 3.2 Evolutionary trade-offs of gene splicing in nuclear-replicating RNA virus

#### 3.2.1 Human immunodeficiency virus type 1

Human immunodeficiency virus type 1 (HIV-1), a lentivirus within the Retroviridae family, remains a devastating global health burden as the causative agent of Acquired Immunodeficiency Syndrome (AIDS). The viral genome comprises two copies of single-stranded positive-sense RNA, which are reverse transcribed into proviral DNA and integrated into the host genome (Luk et al., 2025; Zhang D. et al., 2021). Biologically, HIV-1 presents a fascinating paradox. Despite its compact genome size of approximately 9 kb, it achieves remarkable proteomic diversity via a sophisticated RNA processing landscape. Long-read Nanopore sequencing has recently unveiled the full extent of this complexity, characterizing over 50 distinct splice variants that are subject to strict temporal regulation during infection (Nguyen Quang et al., 2020).

**FIGURE 7:**
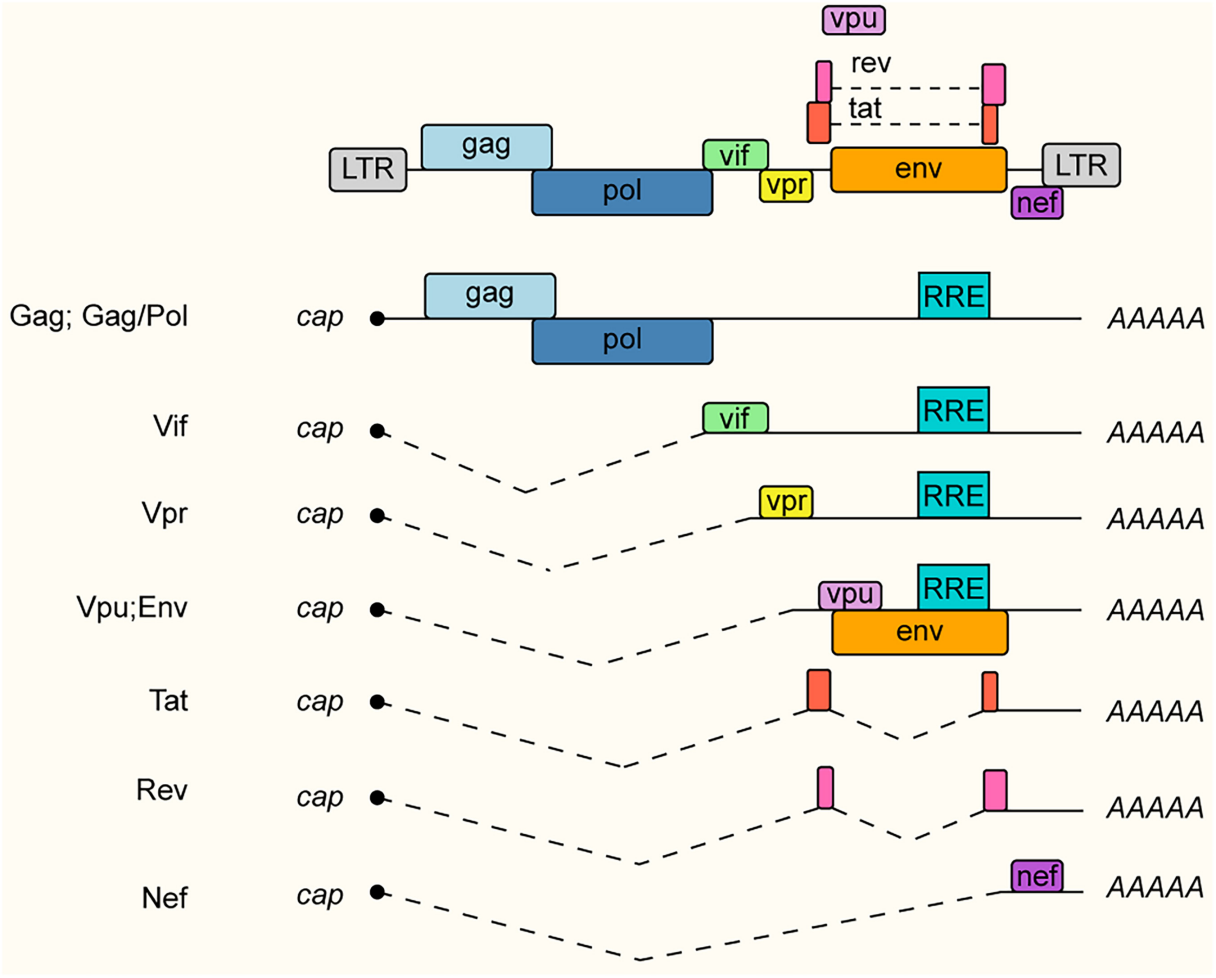
Schematic representation of HIV-1 genomic organization and transcript classes generated via alternative splicing. Transcription is initiated from a single promoter located in the 5′ Long Terminal Repeat (LTR). The full-length transcript undergoes alternative splicing events, indicated by dashed lines connecting splice donor and acceptor sites, to produce three distinct classes of viral RNA. The unspliced RNA (top) functions as the mRNA template for Gag and GagPol polyproteins and serves as the viral genome packaged into progeny virions. Partially spliced transcripts (middle) retain the Rev Response Element (RRE) and encode the envelope protein (Env) and accessory proteins Vif, Vpr, and Vpu, whereas fully spliced transcripts (bottom) accumulate early in replication and encode the regulatory proteins Tat, Rev, and Nef. All transcripts undergo 5′ capping (cap) and 3′ polyadenylation (AAAAA).

This proteomic diversification relies on a strictly balanced three-tiered transcript system derived from a single full-length pre-mRNA. The unspliced transcripts (∼9 kb) primarily function as genomic RNA for virion packaging while also serving as templates for the structural proteins Gag and Pol. A fraction of these transcripts undergoes processing to generate partially spliced mRNAs (∼4 kb), which encode the envelope protein (Env) and accessory factors including Vif, Vpr, and Vpu. Finally, further splicing yields the fully spliced class (∼2 kb), which directs the translation of the regulatory proteins Tat, Rev, and Nef (Figure 7). To circumvent host nuclear surveillance mechanisms that normally sequester intron-retaining transcripts, specifically the unspliced and partially spliced classes, HIV-1 relies on the viral protein Rev. Acting as a molecular adaptor, Rev assembles on the Rev Response Element (RRE) and recruits the host export factor Crm1, facilitating the non-canonical nuclear export of these essential viral RNAs (Pocock et al., 2016; Schimmich et al., 2024).

Crucially, this system hinges on the maintenance of suboptimal splice sites. The artificial optimization of these sites for efficient host recognition would result in the complete processing of all viral transcripts to the fully spliced form, thereby exhausting the genomic RNA pool and abolishing infectivity. This precarious balance renders the splicing machinery a critical vulnerability. Indeed, dysregulated splicing is a defining feature of viral latency; silent reservoirs often actively generate transcripts with aberrant splicing profiles that fail to produce functional proteins (Pasternak and Berkhout, 2018). Consequently, therapeutic strategies targeting this mechanism have emerged as a promising frontier. For instance, the small molecule ABX464 binds to the Cap-Binding Complex (CBC) at the 5′ end of viral RNA to selectively promote splicing efficiency. This pharmacologically induced oversplicing aggressively depletes the essential unspliced genomic RNA required for virion packaging, thereby effectively blocking viral replication and reducing the latent viral reservoir (Vautrin et al., 2019).

#### 3.2.2 Influenza A Virus

**FIGURE 8:**
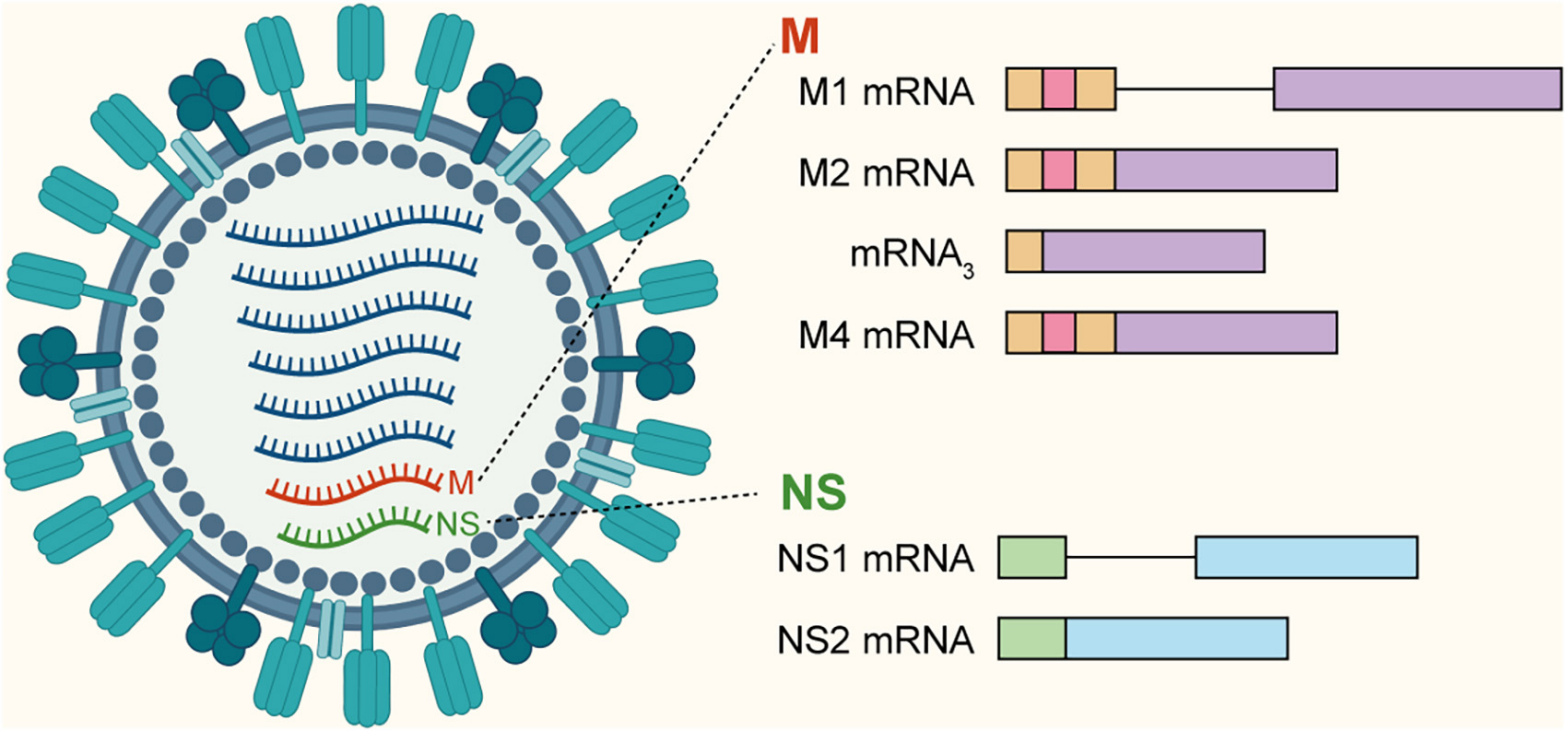
Influenza A Virus mRNA splicing profiles. The viral genome consists of eight segments. Upon transcription, the M and NS mRNAs undergo alternative splicing to generate multiple isoforms. The M segment produces M1, M2, mRNA3, and M4 mRNAs, while the NS segment generates NS1 and NS2 (NEP) mRNAs.

Influenza A Virus (IAV), a prototypical member of the Orthomyxoviridae family, constitutes a persistent and severe global health threat due to its broad host tropism across humans, avian species, and various mammals (Sun et al., 2023; Yang et al., 2022, 2025). Biologically, IAV represents a premier model of viral reliance on host RNA processing. The viral genome comprises eight segments of single-stranded, negative-sense viral RNA (vRNA). To overcome the physical limitations of this highly compact genome, which must efficiently encode at least 10–14 distinct proteins, the virus extensively utilizes alternative splicing (AS), most notably within segments 7 and 8 (Figure 8; Gavazzi et al., 2013; Wang et al., 2014).

Viral segment 7 (the M segment) relies on a delicately balanced splicing mechanism to generate multiple functional proteins from a single precursor. The unspliced transcript encodes the matrix protein (M1), which serves as the primary structural scaffold for the virion, whereas the spliced mRNA yields the M2 ion channel, an essential component for viral uncoating. To align protein production with the high M1:M2 stoichiometric ratio strictly required for virion assembly, the virus purposefully utilizes a “weak” 3′ splice site that is suboptimally recognized by the host splicing machinery (Li et al., 2022; Manzoor et al., 2017; Thompson and Lynch, 2019). This inherent inefficiency ensures that the vast majority of transcripts remain unspliced to support structural assembly, while permitting only a precisely controlled fraction to undergo splicing. Beyond these canonical transcripts, the M segment exhibits further splicing complexity: the M1 mRNA can be alternatively spliced to generate mRNA3, a transcript of currently unknown function, and the M4 mRNA, which encodes an isoform of the M2 protein frequently detected in specific viral strains.

Similarly, segment 8 (the NS gene) exploits AS to yield two functionally distinct proteins. The unspliced transcript is translated into the non-structural protein 1 (NS1), which functions as the paramount antagonist of the host interferon response. Conversely, the spliced mRNA encodes the Nuclear Export Protein (NEP, historically known as NS2), which is responsible for mediating the nuclear export of newly synthesized viral ribonucleoproteins. Notably, this splicing event is subject to strict temporal regulation. The progressive accumulation of the viral polymerase complex during the course of infection shifts the splicing equilibrium to strongly favor NEP production, thereby precisely coordinating the initiation of viral assembly and cellular egress during the late stages of infection (Min and Krug, 2006; Tsai et al., 2025; Zhang X. et al., 2024).

Beyond classical splicing, IAV further expands its coding capacity through alternative translational recoding mechanisms. For instance, PA-X is generated from segment 3 via ribosomal frameshifting and acts as a potent host shutoff factor that aggressively degrades cellular mRNAs (Hayashi et al., 2016; Levene et al., 2021). Meanwhile, PB1-F2, a pro-apoptotic factor encoded by segment 2, is translated from an alternative open reading frame (ORF) via leaky ribosomal scanning (DeLuca et al., 2011; Hohensee et al., 2024). Together with AS, these sophisticated mechanisms allow IAV to encode a remarkably complex functional diversity from a minimalist genetic template.

Collectively, the exploitation of the host splicing machinery represents a universal viral strategy for expanding genomic coding capacity. By generating multiple functional isoforms from restricted genetic templates, viruses effectively circumvent the physical constraints of their compact genomes. This proteomic plasticity not only optimizes gene expression but also enhances adaptability, enabling the precise spatiotemporal coordination of the viral life cycle and the evasion of host innate immunity. Consequently, this absolute dependence on specific, often suboptimal, splicing events exposes an exploitable bottleneck in viral replication, highlighting the viral-host splicing interface as a highly promising target for broad-spectrum antiviral interventions.

## 4 Viral reprogramming of host alternative splicing for immune evasion

Viral infection drives a profound reprogramming of the host cellular environment, necessitating rapid and dynamic alterations in gene expression (Boudreault et al., 2019; Sehrawat and Garcia-Blanco, 2023). While traditional studies have predominantly focused on global transcriptional shifts, comprehensive high-throughput transcriptomic analyses have illuminated that alternative splicing (AS) constitutes a distinct, yet equally pervasive, tier of post-transcriptional regulation during infection (Ashraf et al., 2019). Importantly, this massive splicing response is not merely a passive byproduct of cellular stress; rather, it operates as an active, bidirectional regulatory battleground essential to host-virus interactions (Banerjee et al., 2020). On one hand, the host leverages global AS to rapidly diversify its protective proteome, generating specific protein isoforms required to mount robust antiviral immune responses and maintain metabolic homeostasis (Mahajan et al., 2021; Zhao et al., 2022). A classical paradigm of the host defensive strategy is the isoform switching of the *OAS1* gene. AS of *OAS1* pre-mRNA generates distinct protein isoforms (such as p42 and p46) with divergent C-terminal domains, which dictate their differential subcellular localizations and specific antiviral efficacies against various viral species (Soveg et al., 2021). Similarly, the AS of some genes encoded the key innate immune adaptors and transcription factors, such as *MAVS* and interferon regulatory factors (IRFs), produces truncated or alternative isoforms that intricately fine-tune type I interferon (IFN) signaling (Brubaker et al., 2014; Panthi et al., 2025). Specifically, the MAVS gene encodes a truncated isoform known as miniMAVS, which lacks the N-terminal caspase activation and recruitment domain (CARD) essential for RIG-I sensing. Research demonstrates that miniMAVS functions as a negative regulator by antagonizing full-length MAVS (FL MAVS). By inhibiting FL MAVS-mediated type I IFN production, miniMAVS dynamically modulates the RIG-I-like receptor (RLR) cascade, ensuring a potent antiviral response while preventing excessive, self-damaging immunopathology (Brubaker et al., 2014).

However, invading viruses actively subvert the cellular AS machinery to dismantle host defenses and create a permissive environment for viral own replication (Chen et al., 2024; Valerdi et al., 2021). A striking example of the viral sabotage is orchestrated by the non-structural protein 16 (NSP16) of severe acute respiratory syndrome coronavirus 2 (SARS-CoV-2). Beyond its canonical methyltransferase activity, NSP16 directly binds to the host U1 and U2 snRNAs, thereby globally impairing host mRNA splicing and specifically suppressing the maturation of interferon-stimulated genes (ISGs) (Banerjee et al., 2020). Similarly, IAV deploys a coordinated, dual-pronged strategy to dismantle host gene expression through its NS1 and PA-X proteins. The NS1 protein directly interferes with host pre-mRNA processing by interacting with spliceosomal components and 3′ end cleavage/polyadenylation factors, thereby blocking the maturation and nuclear export of critical antiviral transcripts. Concurrently, the viral endonuclease PA-X triggers a global “host shutoff” by selectively degrading host RNA polymerase II-transcribed mRNAs. Together, these viral effectors synergistically deplete the host’s antiviral transcriptomic pool and redirect the cellular translational machinery toward viral production (Chaimayo et al., 2018; Khaperskyy and McCormick, 2015). Among DNA viruses, the Herpes simplex virus 1 (HSV-1) ICP27 protein exemplifies another sophisticated hijacking strategy. ICP27 physically interacts with the spliceosome and host splicing factors, such as SR proteins, to block host pre-mRNA splicing. Simultaneously, it facilitates the nuclear export of the viral predominantly intronless mRNAs, effectively shutting off host protein synthesis and re-routing cellular resources toward viral production (Chen et al., 2002; Sciabica et al., 2003).

Consequently, the highly altered global splicing landscape observed during infection reflects a dynamic molecular interplay, a direct readout of the ongoing evolutionary arms race that comprises both the defensive countermeasures of the host and the manipulative sabotage strategies of the virus.

## 5 Viral circular RNAs: novel effectors in the host-virus arms race

**FIGURE 9:**
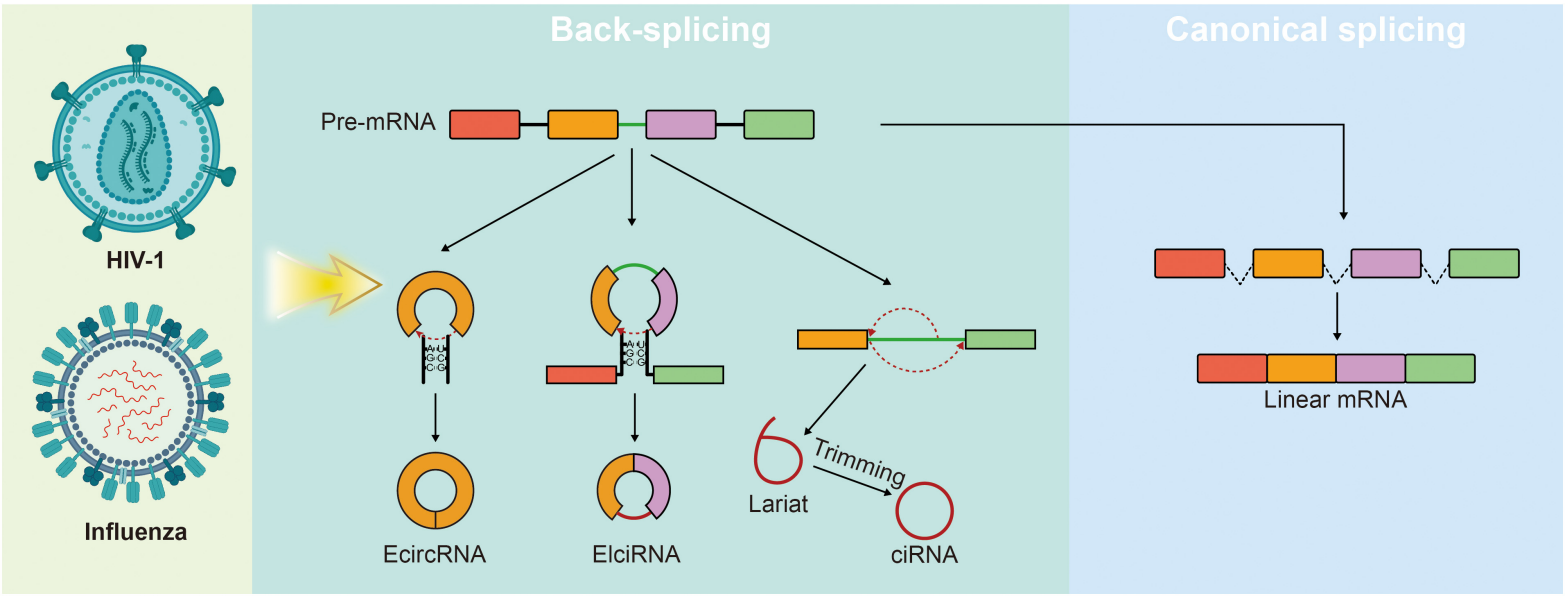
Schematic of circular RNA biogenesis. Unlike canonical splicing that yields linear mRNAs (right), non-canonical splicing generates three distinct circular isoforms (middle). EcircRNAs and EIciRNAs are produced via back-splicing, where a downstream splice donor site is covalently linked to an upstream splice acceptor site. In contrast, ciRNAs are derived from excised lariat introns that escape debranching and undergo 3′-trimming to form stable circular structures.

Distinct from canonical splicing, which joins exons in a linear 5′-to-3′ order, back-splicing is a non-canonical RNA processing event characterized by the covalent linkage of a downstream splice donor site to an upstream splice acceptor site (Figure 9; Gantley et al., 2023). This unique biochemical reaction generates circular RNAs (circRNAs), a class of single-stranded non-coding RNAs featuring a covalently closed loop structure that lacks both 5′ caps and 3′ poly(A) tails (Pitolli et al., 2022; Yang, 2015). Although circRNAs were visualized in electron microscopy studies as early as the 1970s, they were historically dismissed as aberrant splicing by-products or mere “transcriptional noise” (Ju et al., 2022; Stringer et al., 2023). However, the advent of high-throughput sequencing and specialized bioinformatics algorithms has precipitated a paradigm shift in our understanding of these molecules (Qu et al., 2017).

Recent evidence demonstrates that circRNAs are not only abundant and evolutionarily conserved but also exhibit exceptional biological stability due to their inherent resistance to exonuclease-mediated degradation (Enuka et al., 2016; Fontemaggi et al., 2021; Yu et al., 2023). Functionally, host circRNAs have emerged as critical regulators across diverse biological processes, ranging from metabolic modulation and oncogenesis to the fine-tuning of innate immune responses (Liu et al., 2025; Sherafat et al., 2025). Paralleling these discoveries in eukaryotic biology, emerging research has revealed that viral genomes, encompassing both DNA and RNA viruses, also undergo back-splicing to generate viral circRNAs (vcircRNAs) (Tan and Lim, 2021; Yamin et al., 2025). While initially considered accidental artifacts, an increasing number of evidences suggest that these viral non-coding RNAs may exert significant biological functions. In particular, specific vcircRNAs are proposed to act as potent effectors in the host-pathogen arms race, enhancing viral replication by specifically antagonizing host innate antiviral immunity (Liu et al., 2025). A classic example is found in DNA viruses, such as the Epstein-Barr virus (EBV), which encodes highly abundant circular RNAs (e.g., circBARTs). The studies have demonstrated that specific EBV-derived circRNAs can modulate host immune pathways and prevent apoptosis, thereby contributing to immune evasion and viral latency (Ge et al., 2021). Furthermore, the repertoire of viral circRNAs continues to expand with recent breakthroughs in retrovirology. Recent transcriptomic analyses have identified that the human immunodeficiency virus type 1 (HIV-1) also undergoes back-splicing to produce functional vcircRNAs. Given their profound resistance to degradation, these newly discovered HIV-1-derived circRNAs are proposed to persist during the viral life cycle, potentially playing a crucial role in maintaining latent cellular reservoirs and modulating the host environment for long-term persistence (Obi et al., 2026).

### 5.1 The landscape of viral circular RNAs: discovery and identification

The identification of viral circular RNAs (vcircRNAs) was historically impeded by the technical limitations of conventional poly(A)-enrichment protocols, which are inherently biased toward the detection of linear transcripts. The pivotal breakthrough in this field was catalyzed by coupling high-throughput RNA sequencing (RNA-seq) with RNase R digestion, an enzymatic treatment that selectively degrades linear RNAs while preserving covalently closed circular forms.

This methodological advance has unveiled a pervasive landscape of vcircRNAs across diverse viral families (Tagawa et al., 2021). According to the updated VirusCircBase repository, over 60,000 distinct viral back-splicing junction (BSJ) events have been annotated, encompassing a broad spectrum of viral pathogens. This includes large DNA viruses, such as members of the Herpesviridae and Papillomaviridae families, as well as complex RNA viruses, including the Coronaviridae.

Although generally present at lower steady-state abundance compared to their linear counterparts, these circular transcripts exhibit exceptional biological stability and highly tissue-specific expression profiles (Fu et al., 2023). Notably, recent deep sequencing analyses have expanded this catalog to include HSV-1, identifying a novel class of vcircRNAs derived from the latency-associated transcript (LAT) and the immediate-early gene *ICP0*. Crucially, these non-canonical transcripts were previously obscured within the classical viral transcriptome, underscoring a newly recognized layer of viral gene regulation (Dremel et al., 2025).

### 5.2 Biogenesis and functional versatility of viral circular RNAs

Unlike the canonical splicing of linear mRNAs, the biogenesis of vcircRNAs is governed by a highly complex interplay between *cis*-acting elements and *trans*-acting factors. While early models posited that back-splicing was driven primarily by complementary sequences flanking the splice sites, emerging evidence highlights the critical regulatory role of viral modulation (Dhahri and Fondufe-Mittendorf, 2023; Nahand et al., 2020). Viruses actively orchestrate this splicing landscape to favor RNA circularization. For instance, the KSHV RNA-binding protein ORF57 enhances the synthesis of a specific subset of viral and host circRNAs, demonstrating that viruses can specifically co-opt cellular machinery to reshape the circular transcriptome (Dremel et al., 2025). Importantly, recent studies on alpha- and gamma-herpesviruses have unveiled alternative biogenesis pathways entirely distinct from classical spliceosome-mediated back-splicing. In these specific viral contexts, RNA circularization is profoundly resistant to spliceosome perturbation; instead, it relies on host RNA ligases, such as RTCB and RLIG1, to directly catalyze the covalent ligation of viral RNA ends (Dremel et al., 2025).

However, when evaluating the functional relevance of vcircRNAs, it is critical to interpret the existing studies with cautious scrutiny. The circRNA field is currently engaged in intense debates regarding stoichiometry, subcellular localization, and the propensity for artificial overexpression systems to generate non-physiological artifacts. Consequently, the functional validation of vcircRNAs must be subjected to rigorous evidence grading. Definitive functional claims require robust quantification of endogenous expression levels relative to their putative targets, strict validation of back-splicing junctions, and, most importantly, the utilization of reverse genetics to construct back-splicing-deficient mutant viruses for true loss-of-function and rescue analyses during natural infection contexts.

The functional significance of vcircRNAs is proposed to extend far beyond their exceptional structural stability. Many proposed mechanisms, such as the widely cited “miRNA sponge” model, are often extrapolated from host circRNA paradigms. In this model, vcircRNAs are hypothesized to act as competitive endogenous RNAs (ceRNAs) that harbor multiple binding sites for specific host microRNAs. Nevertheless, this mechanism requires strict stoichiometric validation, as endogenous vcircRNA copies must be sufficiently abundant to effectively sequester target miRNAs. To avoid drawing broad claims from potentially artificial systems, the field increasingly relies on deeply supported examples. A paramount example is the Epstein-Barr virus (EBV)-encoded circBARTs. The existence and physiological relevance of circBARTs have been rigorously validated not merely through artificial overexpression, but by utilizing rigorous RNase R enrichment, absolute stoichiometry quantification, and comparative analyses using the EBV B95-8 strain, a natural deletion mutant lacking the BART locus, to unequivocally confirm their origin and roles in viral latency and oncogenesis (Toptan et al., 2018; Ungerleider et al., 2018). Beyond acting as nucleic acid sponges, vcircRNAs have also been suggested to function as sophisticated protein scaffolds or decoys to directly subvert host innate immunity (Ouyang et al., 2016). By physically interacting with key signaling components within the NF-κB or RIG-I pathways, certain viral non-coding RNAs may actively impede signal transduction, thereby dampening the production of interferons and proinflammatory cytokines (Wang W. et al., 2023). Furthermore, emerging evidence implicates vcircRNAs in overarching metabolic reprogramming and cell proliferation, potentially establishing a cellular microenvironment highly conducive to long-term viral persistence and tumorigenesis (Zhou et al., 2020).

### 5.3 Synthetic circRNAs: a new era of antiviral therapeutics

The inherent stability of circular RNAs, stemming from their profound resistance to exonuclease-mediated degradation, has positioned them as a highly promising platform for next-generation antiviral therapeutics and vaccines. Recent bioengineering advances have successfully harnessed back-splicing mechanisms to generate synthetic circRNA vaccines that exhibit superior pharmacokinetic stability and immunogenicity compared to conventional linear mRNA platforms in preclinical models.

Evidence from recent preclinical studies has underscored the efficacy of this platform. For instance, in the context of SARS-CoV-2, a circular RNA vaccine encoding the trimeric Receptor Binding Domain (RBD) elicited potent neutralizing antibodies and durable T-cell responses in non-human primates. Notably, this platform exhibited remarkable thermal stability, thereby streamlining cold-chain logistics and global distribution (Qu et al., 2022). Similarly, a novel circRNA vaccine strategy targeting the Zika virus envelope protein demonstrated high efficacy in preventing infection in animal models without inducing antibody-dependent enhancement (ADE), a critical safety concern historically associated with conventional flavivirus vaccines (Liu et al., 2024).

However, from a future perspective, the clinical application of these technologies must be approached with cautious optimism. While therapeutic use in humans remains largely investigational, several translational hurdles must be systematically addressed before these vaccines can be broadly deployed. These challenges include optimizing large-scale *in vitro* circularization and purification processes, rigorously evaluating long-term safety and tolerability in human cohorts, and comprehensively understanding how synthetic circRNAs might inadvertently trigger host innate immune sensors.

In summary, the characterization of viral circular RNAs represents a paradigm shift in our understanding of viral transcriptomic complexity, elevating what was once dismissed as “transcriptional noise” into a paramount category of functional regulatory molecules. By co-opting the host’s non-canonical back-splicing machinery, viruses generate highly stable transcripts that are increasingly recognized as putative versatile effectors to subvert host innate immunity and reprogram cellular metabolism. This strategy further exemplifies the intense evolutionary pressure on viruses to maximize functional output from compact genomes. Significantly, the physicochemical properties that contribute to viral persistence, specifically inherent biostability, have been successfully repurposed for biomedical innovation. The transition of circular RNAs from agents of pathogenesis to next-generation vaccine platforms highlights the profound transformative potential of studying fundamental viral RNA biology.

## 6 Methodologies for splicing detection and quantification

Accurately characterizing the dynamic splicing landscape is a fundamental prerequisite for understanding viral pathogenesis and host immune responses. However, distinguishing between multiple isoforms that share extensive sequence homology poses significant analytical challenges. Due to the inherent heterogeneity of the transcriptome and the high degree of sequence overlap between transcript variants, the precise absolute quantification of specific isoforms remains technically demanding. Consequently, most analytical frameworks focus on determining the relative ratios of spliced to unspliced transcripts, often expressed as the “percentage spliced in” (PSI) value, rather than absolute copy numbers.

In this section, we categorize current analytical strategies into targeted approaches for precise validation and global profiling methods for high-throughput discovery. We further evaluate their respective strengths and limitations in dissecting the complex viral and host splicing landscapes (Table 1).

**TABLE 1:** Methodologies for splicing detection and quantification.

Category	Methodology	Advantages	Limitations	Primary application
Targeted approaches	Northern blotting	Direct visualization of transcript size; No PCR amplification bias; Detects multiple isoforms simultaneously.	Low sensitivity; High sample input required; Labor-intensive/low throughput	Validation of isoform size and abundance
	RT-PCR/RT-qPCR	High sensitivity and specificity; Broad detection span (qPCR); Cost-effective and accessible.	Requires pre-designed primers; Cannot detect novel isoforms.	Validation of specific exon inclusion or skipping events
	Droplet digital PCR (ddPCR)	Absolute quantification (copy number); High precision without standard curves; Detects low-abundance/rare variants.	Low throughput; Higher cost per sample.	Precise quantification of viral or rare host isoforms
	RNA-FISH	Visualizes subcellular localization; Single-cell resolution.	Low throughput; Technically demanding probe design.	Determining spatial fate of isoforms
Global profiling	Short-read RNA-seq (e.g., Illumina)	High throughput/genome-wide; Deep quantification; Lower cost per base.	Cannot resolve distant exon connectivity; Assembly ambiguity for complex isoforms.	Global discovery of splicing changes and quantification
	Long-read sequencing (PacBio/Nanopore)	Sequences full-length transcripts; Resolves phasing and complex structures; Direct detection of RNA modifications (Nanopore)	Higher operational cost; Requires advanced computational pipelines.	Isoform discovery, phasing analysis, and epitranscriptomics
Functional validation	Targeted mass spectrometry (MS)	Validates protein-level expression; Distinguishes isoform-specific peptides.	Lower sensitivity than RNA methods; Antibody/peptide specificity challenges	Confirmation of translated protein isoforms
	CRISPR/Cas9 engineering	Endogenous context study; Avoids overexpression artifacts.	Potential off-target effects; Time-consuming generation of lines.	Functional characterization and mechanistic studies

### 6.1 Targeted detection of alternative isoforms

Targeted approaches are specifically designed for the precise characterization of specific splicing events. These techniques fundamentally hinge on the hybridization of complementary oligonucleotides to the target mRNA, with specificity governed by the precise binding thermodynamics of the probes or primers used.

Northern blotting, a classical method, entails the electrophoretic separation of total RNA followed by hybridization with labeled probes (Green and Sambrook, 2022). Its primary merit lies in the ability to simultaneously detect multiple transcript variants based on size without introducing PCR amplification bias, thereby providing a direct visual representation of the relative abundance of different isoforms (Ahmad et al., 2021; Ouyang et al., 2019). However, Northern blotting is limited by its low sensitivity, high requirement for input material, and labor-intensive workflow.

Reverse transcription-PCR (RT-PCR) offers a more sensitive and versatile alternative (Huggett, 2020; Weathers et al., 2020). By employing primer sets that span specific exon-exon junctions, researchers can selectively amplify isoforms containing or excluding a cassette exon. While standard end-point PCR yields only semi-quantitative data (Fidalgo et al., 2022), this limitation is overcome by real-time quantitative PCR (RT-qPCR) and droplet digital PCR (ddPCR), which enable high-precision measurement. Notably, ddPCR offers the distinct capability of absolute quantification, which is particularly critical for detecting low-abundance viral transcripts or rare host splice variants (Kojabad et al., 2021; Taylor et al., 2017).

Furthermore, the principle of hybridization extends to RNA Fluorescence *In Situ* Hybridization (RNA-FISH), which visualizes isoforms within their cellular context (Shilo et al., 2022).

This technique is advantageous for determining the subcellular fate, such as nuclear retention versus cytoplasmic export, of specific splice variants. Collectively, while these targeted approaches are unsuitable for broad discovery, they remain the gold standard for validating the presence and ratio of specific isoforms identified by high-throughput sequencing.

### 6.2 Global profiling of splicing landscape

High-throughput RNA sequencing (RNA-seq) has revolutionized the global analysis of AS, enabling the unbiased evaluation of the transcriptome without prior knowledge of specific targets. In contrast to targeted methods reliant on pre-designed primers, standard RNA-seq operates by fragmenting RNA and sequencing millions of short reads to computationally reconstruct the transcriptome (Hong et al., 2020; Kukurba and Montgomery, 2015). This approach offers a comprehensive, genome-wide snapshot, facilitating the discovery of novel isoforms and the simultaneous quantification of splicing changes across thousands of genes (Jaganathan et al., 2019; Zhang et al., 2019). However, short-read sequencing is inherently limited in resolving complex isoforms. Specifically, short reads often fail to resolve long-range exon connectivity (phasing) between distant exons, leading to potential inaccuracies in full-length transcript assembly (Jin et al., 2025; Zhang et al., 2023).

This limitation is increasingly addressed by long-read sequencing technologies, such as Pacific Biosciences (PacBio) and Oxford Nanopore Technologies (ONT), which sequence full-length transcripts from the 5′ cap to the 3′ poly(A) tail (Kabza et al., 2024). Notably, Nanopore sequencing offers the distinct advantage of direct native RNA sequencing, allowing for the simultaneous detection of RNA modifications alongside splicing patterns, thereby revealing crucial layers of post-transcriptional regulation (Jenjaroenpun et al., 2021; Liu et al., 2019). Despite its transformative potential, long-read sequencing currently faces several notable limitations that must be critically considered. First, platforms like Oxford Nanopore inherently exhibit higher per-base error rates compared to short-read sequencing, often necessitating hybrid error-correction strategies combined with highly accurate Illumina reads. Second, both the relatively high cost per run and the requirement for substantial amounts of high-quality input RNA impede its widespread application, particularly when dealing with some critical clinical samples contained low viral loads. Finally, the sheer volume and structural complexity of long-read data introduce significant computational challenges. Resolving intricate viral splicing events, dynamic isoform switching, and overlapping transcripts demands specialized bioinformatics pipelines and substantial computational infrastructure.

Therefore, while long-read technologies offer unparalleled transcript-level resolution, their current optimal utility often relies on synergistic integration with short-read datasets. Consequently, bioinformatic predictions from these platforms still necessitate subsequent validation via targeted RT-PCR or functional assays to ensure biological relevance.

### 6.3 Validation and functional characterization of AS event

Bioinformatic identification of AS signatures is merely the first step. These predictions must be substantiated through rigorous experimental validation. A systematic experimental framework typically entails a multi-tiered approach satisfying three core criteria: (1) orthogonal validation of transcription isoforms, (2) confirmation of corresponding protein variants, and (3) demonstration of causality via targeted *cis*- or *trans*-regulation (Mann et al., 2023).

While RNA-seq defines the global transcriptome, validating the specific inclusion or exclusion of exons requires orthogonal methods such as semi-quantitative or quantitative RT-PCR (Rebs et al., 2022). These techniques are essential for verifying the precise splicing ratios derived from sequencing data, particularly across different cell types or infection conditions (Ueda et al., 2024). However, transcript presence does not guarantee protein translation. Characterizing endogenous protein isoforms remains a significant bottleneck due to low expression levels and the high sequence homology between isoforms, which frequently compromises the specificity of antibody-mediated detection (Fan et al., 2024).

To overcome these limitations, targeted mass spectrometry (MS) has emerged as a powerful tool for distinguishing isoform-specific peptides (Liu et al., 2017). Alternatively, precise genome engineering, such as CRISPR/Cas9-mediated editing, can be employed to introduce epitope tags or generate isoform-specific knockouts. This enables the study of endogenous protein dynamics without the artifacts classically associated with ectopic overexpression (Esmaeili Anvar et al., 2024).

To definitively link an AS event to a specific phenotype, it is critical to distinguish splicing regulation from other post-transcriptional processes, such as mRNA stability or translational control (Baralle and Giudice, 2017). Mechanistic dissection can be achieved by modulating specific RNA-binding proteins (RBPs), identified via motif analysis or CLIP-seq, or by directly targeting *cis*-acting elements using antisense oligonucleotides (ASOs) (Jacob et al., 2024). Finally, given that immortalized cell lines often exhibit dysregulated splicing profiles distinct from their tissues of origin, it is critical to validate key observations in primary cells or relevant *in vivo* animal models to ensure physiological relevance, particularly in the context of viral infection and oncogenesis (Tripathi et al., 2017).

Ultimately, the transition from identifying a splicing event to elucidating its biological function requires an integrative workflow. By combining high-resolution transcriptomic validation with precise protein characterization and mechanistic dissection, researchers can robustly distinguish functional isoforms from incidental transcriptional noise. This rigorous validation strategy is indispensable for establishing specific viral splicing events as viable therapeutic targets and for accurately defining their roles in viral pathogenesis.

## 7 Conclusion and future perspectives

The interplay between viral pathogens and the host RNA splicing machinery represents a sophisticated evolutionary adaptation, driven by the stringent constraints of compact viral genomes. As highlighted herein, viruses ranging from the segmented Influenza A Virus to DNA viruses such as HBV and HPV have evolved convergent strategies to exploit the host spliceosome (Ashraf et al., 2019; Schwartz and Ast, 2010). By hijacking this cellular machinery, viruses achieve two critical objectives: maximizing the coding potential of restricted genetic templates and ensuring the precise spatiotemporal regulation of viral gene expression. However, this absolute dependency constitutes a strategic vulnerability. While alternative splicing endows viruses with the proteomic plasticity required for replication and persistence, their reliance on suboptimal splice sites and specific host splicing factors exposes an exploitable bottleneck that is highly amenable to therapeutic intervention.

Furthermore, the discovery of vcircRNAs has fundamentally expanded our understanding of viral RNA processing. The recognition that diverse viruses utilize non-canonical back-splicing to generate highly stable, immunomodulatory transcripts challenges the traditional dogma that viral transcriptomes are exclusively linear. These covalently closed circular molecules, functioning as potent miRNA sponges or protein decoys, add an unprecedented layer of complexity to the virus-host interactome, particularly in the context of innate immune evasion. Significantly, the inherent biostability that renders these molecules effective viral effectors is now being actively harnessed to develop next-generation circular RNA vaccines, exemplifying the rapid translation of fundamental viral RNA biology into transformative biomedical innovation (Qu et al., 2022).

Looking forward, the integration of long-read nanopore sequencing with advanced targeted proteomic approaches is poised to unveil the full, unannotated spectrum of viral splicing isoform diversity and their distinct functional roles in pathogenesis (Depledge et al., 2019). Future research must prioritize dissecting the highly specific molecular crosstalk between cis-acting viral RNA elements and trans-acting host splicing factors to identify novel, druggable interfaces. From a translational perspective, this virus-host splicing axis offers a compelling avenue for the development of splicing-targeted antivirals. Several small-molecule splicing modulators, such as inhibitors targeting the SF3b complex or SR protein kinases (SRPKs), have already demonstrated potent broad-spectrum antiviral efficacies *in vitro* (Fukuhara et al., 2006; Kyei et al., 2018; Li et al., 2022). However, the clinical translation of these modulators faces a formidable challenge: achieving absolute viral specificity without inducing severe host toxicity. Because global pre-mRNA splicing is indispensable for fundamental host cell viability, broad-spectrum splicing inhibitors inherently possess a narrow therapeutic window. To overcome this toxicological hurdle and provide a robust translational outlook, future therapeutic paradigms must pivot toward precision targeting. Emerging precision RNA-targeting technologies, such as steric-blocking ASOs or programmable RNA-targeting CRISPR-Cas systems, hold immense promise (Abbott et al., 2020; Freije et al., 2019). By rationally designing for specifically blocking viral splicing sites or disrupting uniquely hijacked viral-host protein interfaces, it may be possible to inhibit viral replication while minimizing the impact on the global host transcriptome. Ultimately, by precisely exploiting these highly conserved cellular dependencies rather than targeting mutable viral enzymes, such next-generation strategies inherently possess a higher genetic barrier to viral resistance compared to conventional direct-acting antivirals.
